# Supplementary figures and images for: Encoding of social exploration by neural ensembles in the insular cortex
Source: PLoS Biol. 2020 Sep 21;18(9):e3000584. doi: 10.1371/journal.pbio.3000584 (PMC7529241; doi:10.1371/journal.pbio.3000584)

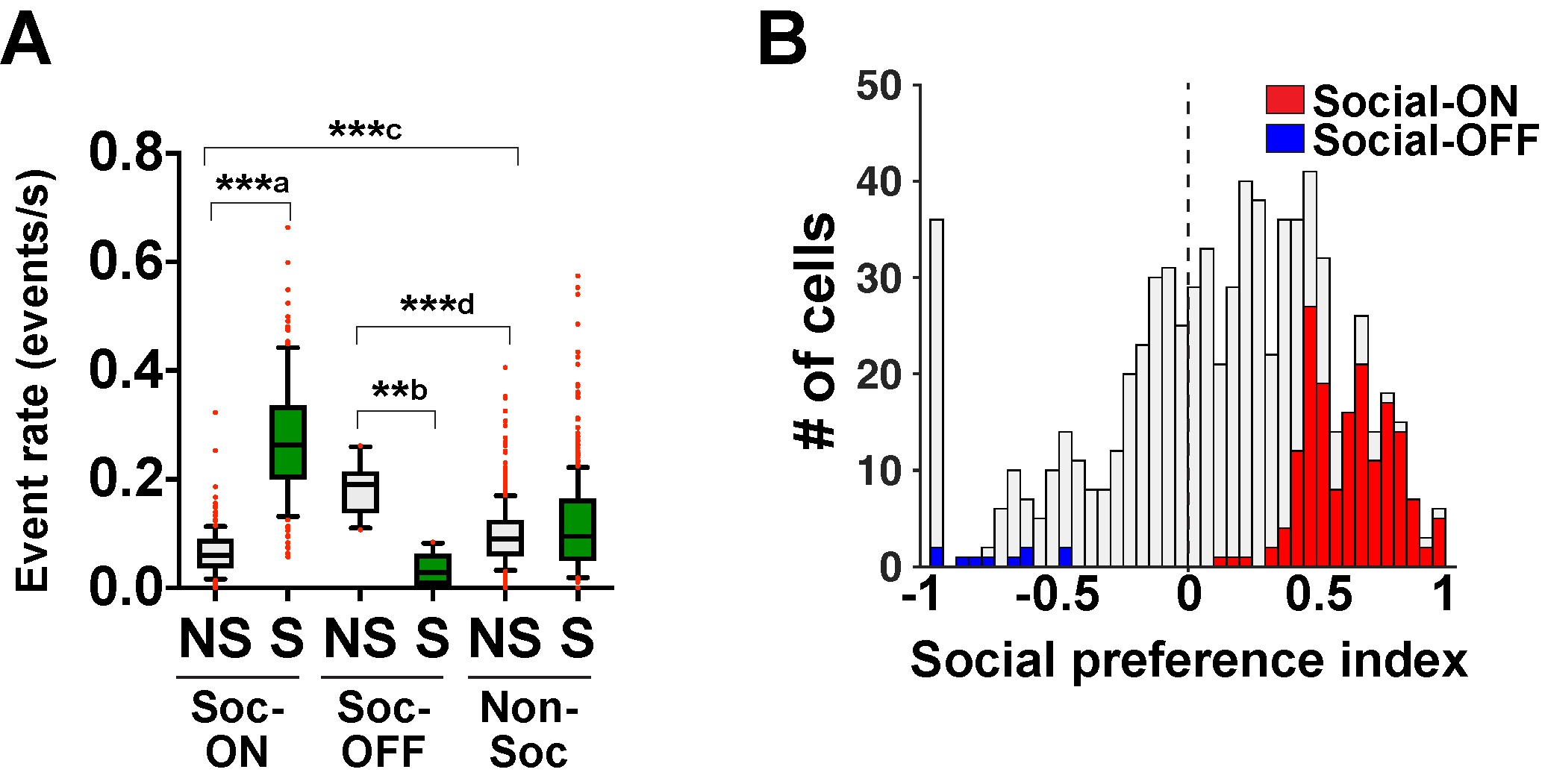

Supplement: S1 Fig — (A) Box plots of Ca2+ event rates of Social-ON (“Soc-ON,” n = 168 cells), Social-OFF (“Soc-OFF,” n = 10 cells), and nonsocial cells (“Non-Soc,” n = 559 cells) during nonsocial periods (NS) and social interaction periods (S). Whiskers represent 10–90 percentile, and red dots represent outliers. ***a, P < 0.0001, W(168) = 14,196, n = 168 cells; **b, P = 0.0020, W(10) = −55, n = 10 cells; Wilcoxon matched-pairs sign rank test; ***c, P < 0.0001, U(168, 559) = 30,636; n = 168 and 559 cells; ***d, P < 0.0001, U(10, 559) = 673; n = 10 and 559 cells; Mann-Whitney test (S1 Data, sheet S1A Fig). (B) Distribution of social preference indices of individual neurons. The fractions of Social-ON cells, Social-OFF cells, and nonsocial cells are shown in red, blue, and gray in stacked bars (S1 Data, sheet S1B Fig). (TIF) [file pbio.3000584.s001.tif]

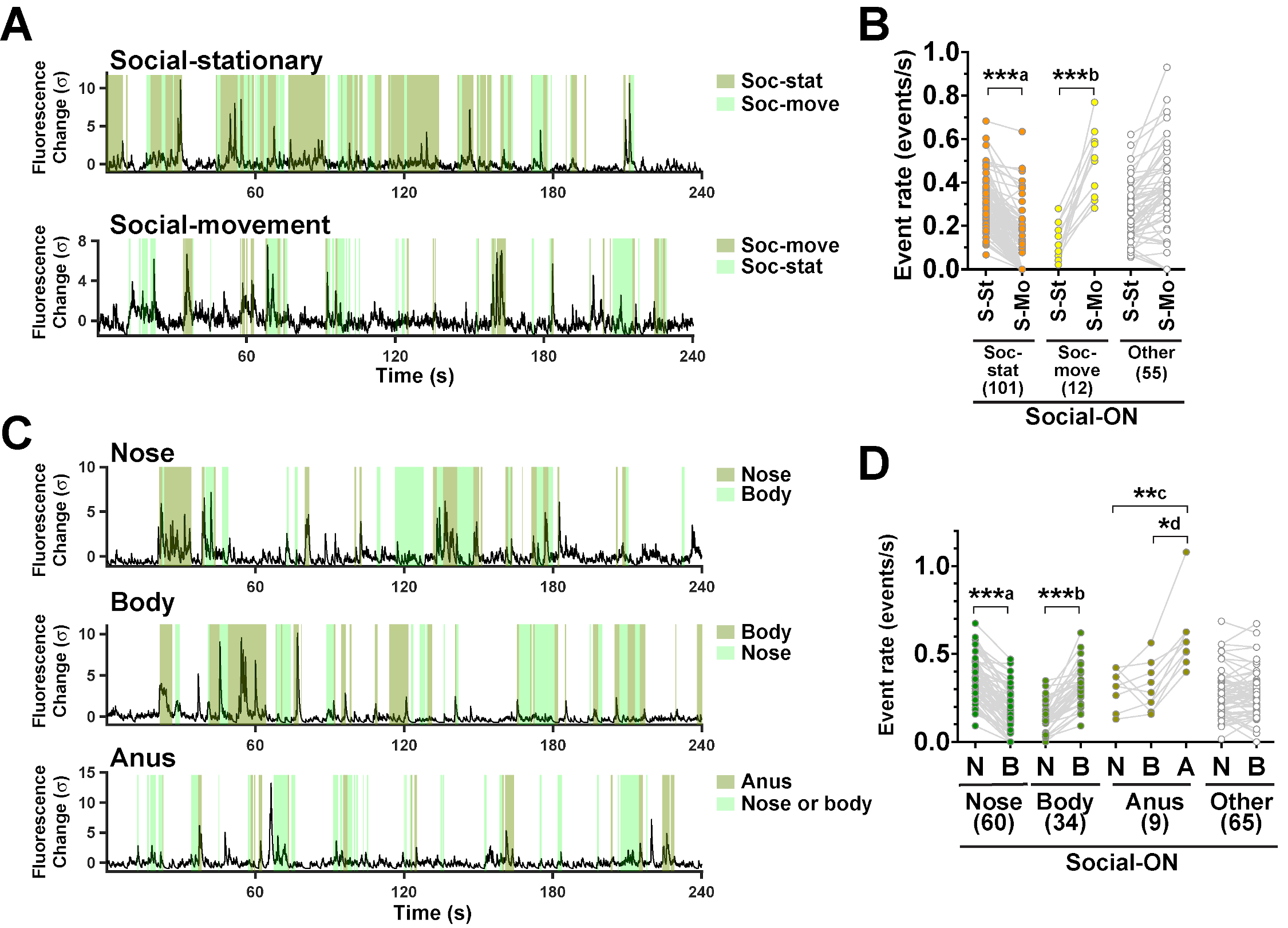

Supplement: S2 Fig — (A) GCaMP6f fluorescence change of a Social-stationary cell (top) and a Social-movement cell (bottom). (B) Ca2+ event rates of Social-stationary cells (“Soc-stat”), Social-movement cells (“Soc-move”), and other Social-ON cells (“other”) during social interaction with (“S-Mo”) and without (“S-St”) movement. ***a, P < 0.0001, W(101) = 5,075; ***b, P = 0.0005, W(12) = −78; Wilcoxon matched-pairs sign rank test (S1 Data, sheet S2B Fig). (C) GCaMP6f fluorescence change of nose (top), body (middle), and anus (bottom) subtypes of Social-ON cells. (D) Ca2+ event rates of nose, body, anus, and other subtypes of Social-ON cells during social interaction with contact with nose (“N”), body (“B”), and anus (“A”). Since the fraction of time spent contacting anus was low, only event rates during contact with nose and body are shown for nose, body, and other cell subtypes. ***a, P < 0.0001, W(60) = −1,830; ***b, P < 0.0001, W(34) = 595; Wilcoxon matched-pairs sign rank test.; **c, P = 0.0012 versus N; *d, P = 0.014 versus B; Friedman test with Dunn’s multiple comparisons test (S1 Data, sheet S2D Fig). (TIF) [file pbio.3000584.s002.tif]

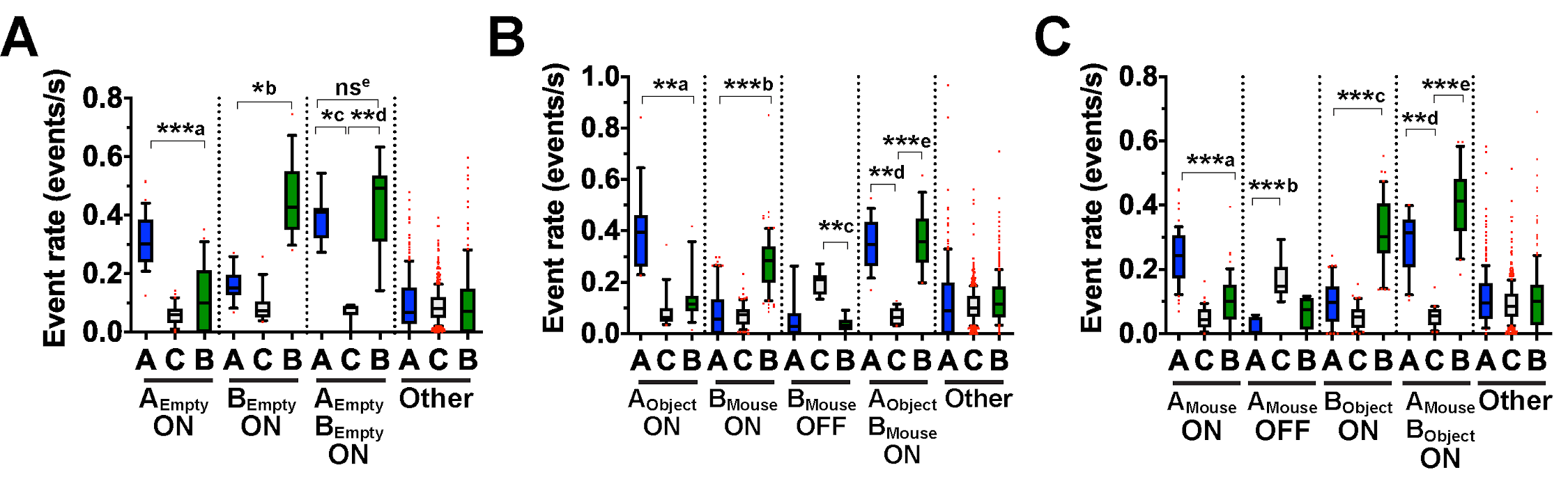

Supplement: S3 Fig — (A) Box plots of Ca2+ event rates of Chamber A-ON cells (AEmpty-ON, n = 32 cells), Chamber B-ON cells (BEmpty-ON, n = 13 cells), Chamber AB-ON cells (AEmptyBEmpty-ON, n = 7 cells), and other cells (Other, n = 527 cells) during the periods when the subject mice investigated Chamber A (“A”), Chamber B (“B”), or otherwise (“C”) in control sessions. Whiskers represent 10–90 percentile, and red dots represent outliers. Cell categories whose fractions are larger than 1% are shown. ***a, P < 0.0001; *b, P = 0.018; *c, P = 0.023; **d, P = 0.0099; nse, P > 0.99; Friedman test with Dunn’s multiple comparisons test (S1 Data, sheet S3A Fig). (B) Box plots of Ca2+ event rates of Chamber A-ON cells (AObject-ON, n = 16 cells), Chamber B-ON cells (BMouse-ON, n = 71 cells), Chamber B-OFF cells (BMouse-OFF, n = 9 cells), Chamber AB-ON cells (AObjectBMouse-ON, n = 14 cells), and other cells (n = 470 cells) in the first interaction session. **a, P = 0.0044; ***b, P < 0.0001; **c, P = 0.0096; **d, P = 0.001; ***e, P < 0.0001; Friedman test with Dunn’s multiple comparisons test (S1 Data, sheet S3B Fig). (C) Box plots of Ca2+ event rates of Chamber A-ON cells (AMouse-ON, n = 59 cells), Chamber A-OFF cells (AMouse-OFF, n = 8 cells), Chamber B-ON cells (BObject-ON, n = 40 cells), Chamber AB-ON cells (AMouseBObject-ON, n = 21 cells), and other cells (n = 451 cells) in the second interaction sessions. ***a, P < 0.0001; ***b, P = 0.0009; ***c, P < 0.0001; **d, P = 0.0021; ***e, P < 0.0001; Friedman test with Dunn’s multiple comparisons test (S1 Data, sheet S3C Fig). (TIF) [file pbio.3000584.s003.tif]

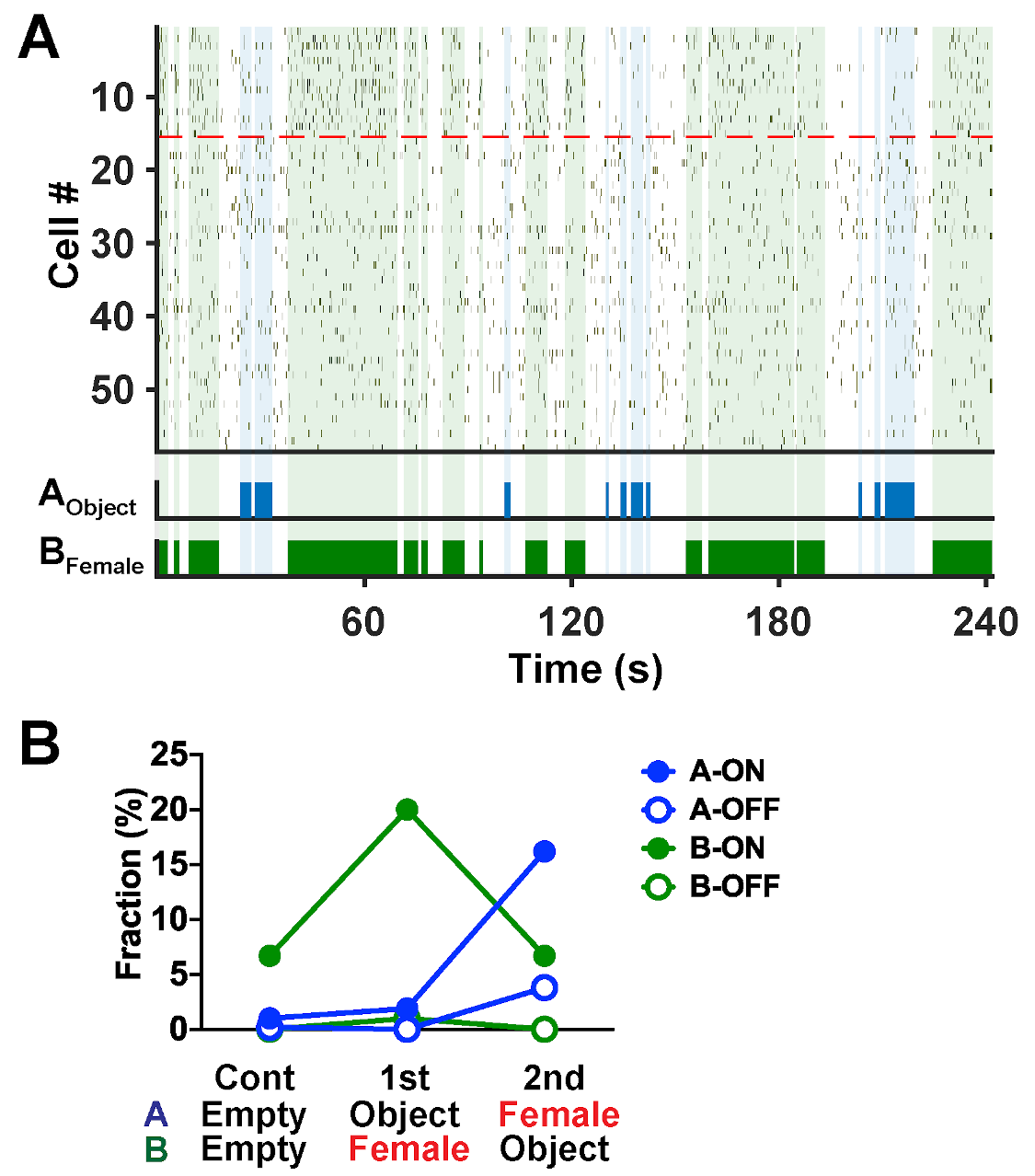

Supplement: S4 Fig — (A) A raster plot showing Ca2+ events of a population of AI neurons (n = 61 cells) imaged in a single experiment during the first interaction session with a female stranger. BFemale-ON cells are sorted above the red dashed lines. The epochs of nose poking to chamber A with a novel object (AObject) and chamber B with a female stranger (BFemale) are shown in the bottom panel and indicated by blue and green shades, respectively. (B). Changes in the fractions of A-ON, A-OFF, B-ON, and B-OFF cells across sessions (n = 105 cells from 2 mice; S1 Data, sheet S4B Fig). The content of each chamber is shown at the bottom. (TIF) [file pbio.3000584.s004.tif]

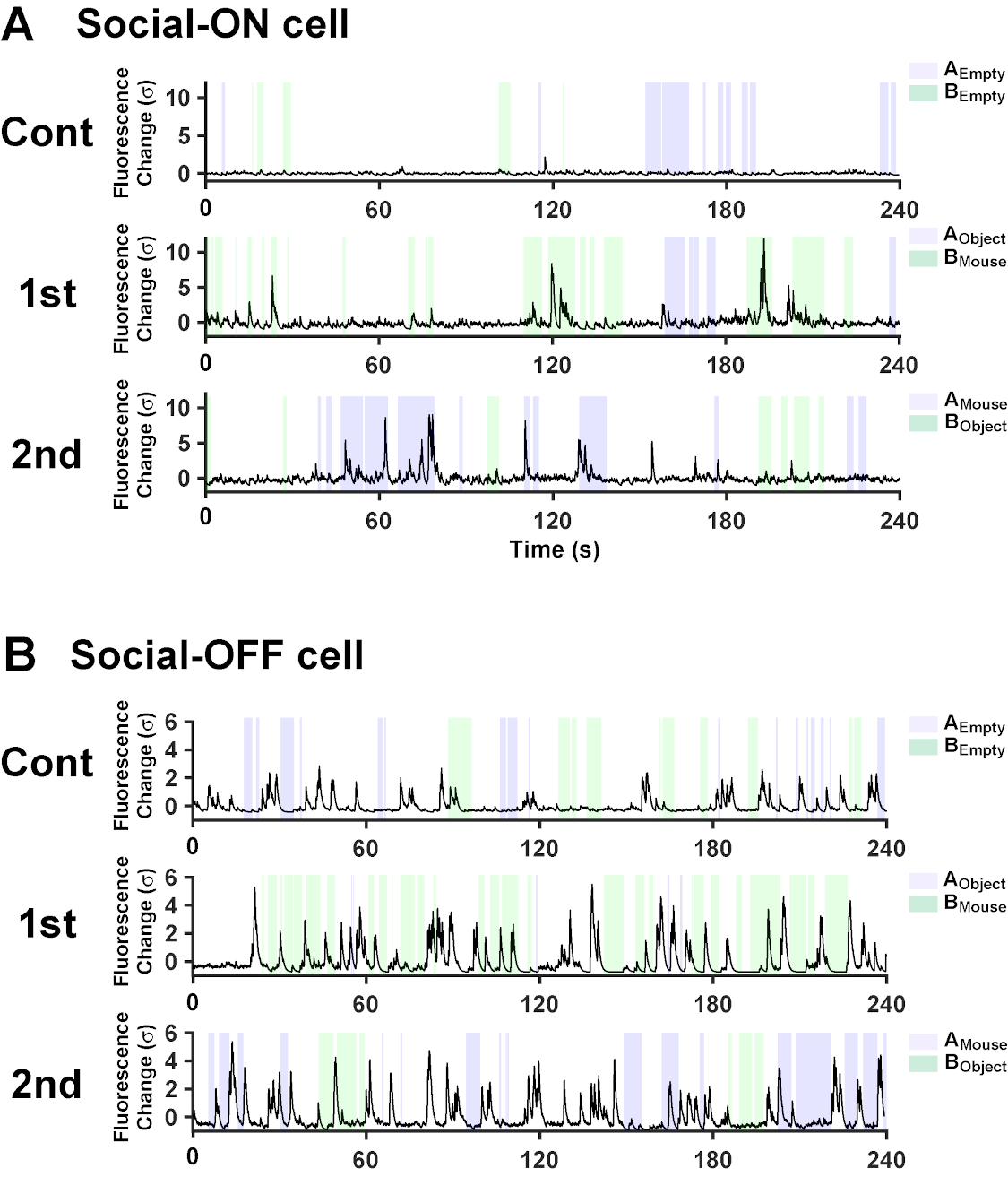

Supplement: S5 Fig — (A). GCaMP6f fluorescence change of a Social-ON cell during control (top, “Cont”), first interaction (middle, “1st”), and second interaction sessions (bottom, “2nd”) of LC experiments. (B) GCaMP6f fluorescence change of a Social-OFF cell during control (top), first interaction (middle), and second interaction sessions (bottom) of LC experiments. (TIF) [file pbio.3000584.s005.tif]

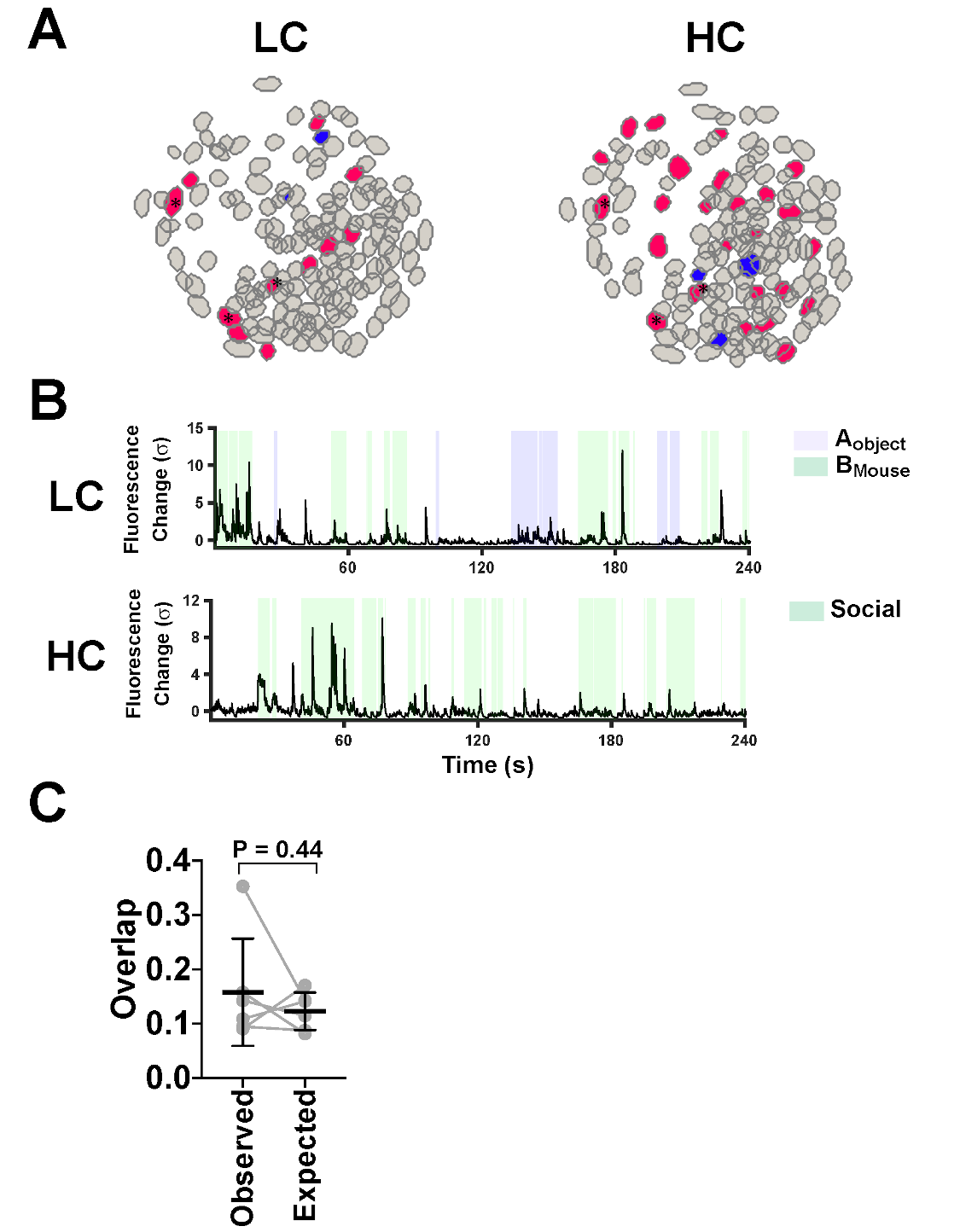

Supplement: S6 Fig — (A) Example social cell maps of LC experiments (left) and HC experiments (right) imaged in the same animal. BMouse-ON cells and BMouse-OFF cells in the first interaction session of LC experiments and Social-ON cells and Social-OFF cells in HC experiments are shown in red and blue, respectively. The ON cells common to the two paradigms (common Social-ON cells) are indicated by asterisks. (B) GCaMP6f fluorescence change of a common Social-ON cell during the first interaction session of LC experiment (top) and HC experiment (bottom). (C) Overlap of the ON cell ensembles observed between HC experiments and LC experiments (“Observed”) and that expected by chance (“Expected”) (paired t test, n = 6 mice; S1 Data, sheet S6C Fig). (TIF) [file pbio.3000584.s006.tif]
