# Supplementary material for: Encoding of social exploration by neural ensembles in the insular cortex
Source: PLoS Biol. 2020 Sep 21;18(9):e3000584. doi: 10.1371/journal.pbio.3000584 (PMC7529241; doi:10.1371/journal.pbio.3000584)
Supplement: Attachment — Submitted filename: response200516.docx [file pbio.3000584.s009.docx]

**Point-by-point responses to the reviewers’ comments**

We thank the reviewers for their highly constructive comments. Reviewer #1 commented, “*this manuscript has potential significance and novelty to be suitable for publication in PLOS Biology*”. Reviewer #2 mentioned, “*the novelty and relevance of this report is potentially very high*” and “*the new data can be useful to shed light on the contribution of insula to social behavior/cognition*”. Finally, Reviewer #3 also mentioned “*the finding of the AI cells with activity is interesting*”. In view of these supportive comments, we extensively revised the manuscript to address all issues raised by the reviewers. Most notably, we re-analyzed our calcium imaging data using the new algorithm CNMF-E (Constrained Nonnegative Matrix Factorization for microEndoscopic data; Zhou et al., 2018; Giovannucci et al., 2019) to deal with the technical concerns associated with the previous image analysis and thoroughly revised the text, most notably the Discussion, so that our findings are readily accessible and discussed in relation to recent relevant literature.

We highlighted all relevant changes in the revised text in red. We sincerely hope that these changes alleviate the reviewers’ concerns and that they find our manuscript suitable for publication in *PLOS Biology*.

*Reviewer #1: Miura et al. employed microendoscopic calcium imaging to study the social behavior related neural ensembles in the agranular insular cortex (AI). The authors found that the activity of two neural ensembles, a larger ensemble of Social-ON neurons and a smaller ensemble of Social-OFF neurons, changed in opposite directions during social exploration. More specifically, Social-ON cells encode social investigation independent of location and were preferentially active during exploration. Encoding of social behavior in the AI is an important subject that is relevant to many psychiatric disorders. Therefore, this manuscript has potential significance and novelty to be suitable for publication in PLOS Biology. However, first and foremost, the authors need to revisit their data analysis. It has been shown in the literature that CNMFe algorithm outperforms PCA/ICA algorithm in many regards. The authors should reanalyze their data using the now widely accepted CNMFe algorithm. In the identification of behavior relevant neurons, the authors need to consider correction for multiple comparison, instead of simply use “greater than the 95th percentile and smaller than the 5th percentile of the values obtained form the randomly permuted data”.  In its current form, the results in the manuscript are descriptive. The authors didn’t provide sufficient quantitative analyses to support their claims. It is this reviewer’s opinion that this manuscript, in its current form, is not suitable for publication in PLOS Biology. Below are some examples of major concerns and comments:*

In light of this comment, we re-analyzed our imaging data using CNMF-E as suggested (page 25, lines 385–390). The use of CNMF-E increased the number of cells extracted from movies by 1.53-fold (from 482 to 737 cells from 9 mice). In addition, we corrected the statistical significance level for multiple comparisons in the identification of behaviorally-tuned neurons (page 26, line 413–page 27, line 429). We confirmed that our main findings and conclusions in the previous version of the manuscript are valid after this extensive and more rigorous data re-analysis.

*1. Figure 1
(a) Figure 1 and FigureS1 should be combined to help reader to understand the results. Figure S1 lacks scale information for both field of view (x-y axis) and calcium traces (amplitude). The signal to noise ratio of the calcium traces seems low from Figure S1B. This reviewer would suggest the authors to add representative calcium images or raw (not heavily processed) calcium video to the manuscript, to convince the readers that some of the cells are not falsely detected by the cell identification algorithm.*

Following this suggestion, we now present the previous Figure S1 as a part of the main figures. More specifically, we have combined it with Fig 2 rather than with Fig 1, as we found that it made the manuscript flow more naturally (Fig 2A–C; page 40, line 647–page 41, line 657).

In this new Fig 2, we have revised Fig 2B (previously Figure S1B) using the data obtained from our CNMF-E analysis and have also added the missing information on the scale of the field of view and response amplitude to it (Fig 2B). Furthermore, we have presented a raw calcium imaging video as S1 Movie, as the reviewer suggested (S1 Movie; page 6, line 66; page 48, lines 780–782).

To ensure that the cells we identified using CNMF-E are true cells, we visually inspected all candidate cells in ∆F/F movies and manually rejected those that did not show appropriate cell morphology and fluorescence intensity changes. We have explicitly stated this procedure in Materials and Methods (page 25, lines 388–390).

*(b) Figure 1B indicates that mice did not explore the novel object at all*.

We thank the reviewer for pointing this out. Fig 1B represents the total duration and number of nasal contacts with the interaction target. The mice therefore appear not to explore the object unless they made direct contact to it.

To address this comment, we analyzed the behavior of the mice during control sessions in more detail. Careful examination of the videos revealed that the mice often sat at a distance from the object and looked and sniffed towards it as if they were cautious about it. In our quantitative analysis, the mice spent 14.0 ± 9.5% and 86.0 ± 9.5% of total time (mean ± s.d., n = 9 mice) in the halves of the cage with and without the object, respectively, and showed sniffing behavior towards the object 13.1± 6.5 times (total duration, 58.7 ± 22.5 s, equivalent to 24.5 ± 9.4% of total time). These results suggest that the mice had their interest in the object, although they were physically remote from it. To explicitly indicate this point, we have included a part of these results in the text (page 6, line 54–56).

*(c)  Rearrange the panels of current Figure 1 A, B, E, D, C to new A, B, C, D, E in revised Figure1 may help readers to follow the information flow of Figure 1 better.*

Following this comment, we have rearranged Fig 1 as suggested (Fig 1; page 39, lines 629–631, page 40, lines 638–645).

*(d) It is difficult to tell the differences between social cells (above the red line) and non-social cells (below the red line) from Figure 1C. This would make the findings of this manuscript questionable*

We apologize for this ambiguity in our data presentation. Although our data presented in Fig 2F and S1 Fig clearly show that our analysis successfully identified a population of Social cells, we understand that the presentation of a particular raster plot can be affected by the pixel resolution of the figure and the extent of background activity. To address this concern, we have presented another example plot in Fig 1E that more clearly shows the increase of activity of Social-ON cells during social interaction (Fig 1E).

2*. Figure 2,3
(a) In my personal opinion, it is not necessary to show Figure 2D-E, figure 3E-I. For instance, the Social-ON cells are supposed to have higher activation rate in social time than that in non-social time. Because this is the way that the authors define (identify) them*.

We thank the reviewer for this suggestion. We assume that the reviewer referred to the previous Figure 3E, 3G and 3I by “figure 3E-I”. While we admit some redundancy among the data the reviewer referred to, we believe that the group data of event rates shown in the previous Figures 2D, 3E, 3G, and 3I and social preference of individual cell activity shown in the previous Figure 2E are important for readers to validate our identification of relevant cell categories. We have thus moved the previous Figure 2D-E and the previous Figure 3E, 3G and 3I to S1 Fig and S3 Fig, respectively (S1 Fig and S3 Fig; page 44, lines 705–714; page 45, line 736–page 46, line 753).

*(b) Figure 2F needs to add scale bar and change the unit in pixels to actual anatomical scale.*

Following this comment, we have added a scale bar to Fig 2G (formerly Figure 2F) and changed the unit of distance to µm. (Fig 2G; page 41, line 668)

*(c) After removing repetitive data from these two figures, Figure2-3 only provide some basic descriptive observations (i.e., number of different type of cells). More mechanistic insights regarding AI cells in coding social behavior need to be provided.*

We thank the reviewer for this constructive comment. Although we agree that providing mechanistic insights would ideally strengthen the significance of this study, we consider that it is beyond the scope of this Short Report manuscript, as the Editor also mentioned “*we will not require further mechanistic insights for publication in our journal*“ in the decision letter.

In response to this reviewer comment, however, we have instead added a discussion on possible future experiments, which reads “*Thus, future studies such as identification and manipulation of the projection targets of Social-On and Social-OFF cells and comparison of their activity between anterior and posterior ICs in a socioemotional task will greatly advance our understanding of their significance and circuit mechanisms.*” (page 15, line 225–page 16, line 228).

*4. Figure 4. Through this figure together with Figure S4, the authors were trying to find the consistent social cells. Unfortunately, the authors fail to provide any quantitative analysis to support their claim. Moreover, the cell numbers are too low to differentiate from randomness. There is no information in the manuscript detailing how the authors registered the cells in two different experiments in the manuscript, related to Figure S4A in which the two neural maps look quite different, raising concerns about cell registration process across different imaging sessions.*

Regarding cell registration procedures, cells in linear-chamber (LC) experiments (Figs 3 and 4) were identified from single concatenated movies that spanned the control, the first interaction and the second interaction sessions. Therefore, all the cells were completely registered across these sessions. We have explicitly stated this information in Materials and Methods (page 25, lines 390–392). For comparisons of cell ensembles between home-cage (HC) experiments and LC experiments, we have now registered cellular maps obtained from these different experiments using the longitudinal registration function provided by Inscopix Data Processing Software (page 28, lines 444–448). This method implements automatic identification of the same neurons across different sessions by taking into account the spatial correlation of individual cell images and has been used in published studies (e.g. Zhang et al., Neuron, 2020, ref [56]). The use of this algorithm allowed us to quantitatively assess the degree of cell registration in this analysis. We found that 70.5% (432 of 613 cells from 6 mice) and 74.5% (432 of 580 cells from 6 mice) of cells identified in HC experiments and LC experiments, respectively, were registered with their counterpart cells in the other map. We have included this information in the text (page 28, lines 448–451).

We then conducted quantitative analyses on the persistence of Social cells within LC experiments and between HC experiments and LC experiments. The results indicate that the number of persistent Social-ON cells observed across different sessions in LC experiments is significantly larger than that expected by chance (Fig 4D; page 13, lines 179–182; page 29, lines 459–462; page 43, lines 700–703). By contrast, the extent of the overlap of Social-ON cell ensembles between HC experiments and LC experiments did not significantly differ from the chance level (S6C Fig; page 13, line 190–page 14, line 195). These results imply that the consistency of ensembles may be altered according to behavioral tasks, contexts and/or time elapsed.

*5. Figure S2BDF: no y-axis label*

We have added y-axis labels to S2B Fig, S2D Fig and S2F Fig accordingly. (S2B Fig, S2D Fig and S2F Fig).

*Reviewer #2: This manuscript reports on an experiment in which the activity of mouse agranula insular cortex CamKII neurons was quantified during a social behavior task using in vivo GCAmp and miniature endoscopes (miniscope). This is an understudied area of the brain that very likely plays important roles in social and emotional behavior and the use of miniscopes in this region is a first, to my knowledge in a social behavior setting. Thus the novelty and relevance of this report is potentially very high. It seems that the experimental procedures and analysis of miniscope data are consistent with the standard in the field and the new data can be useful to shed light on the contribution of insula to social behavior/cognition; however the manuscript discussion makes many speculations that are well beyond the supporting data, and the key conclusion, that insula provides a sort of online status of social interaction (which seems to be supported by data) is not well framed in the discussion which diminishes enthusiasm for the paper (see below).

The authors must also consider reducing the number of cell groups discussed. Several paragraphs of results name the number of cells that fall into different categories, accounting for only a very tiny fraction of observed neurons (i.e. 0.8% of cells were anticorrelated with a specific chamber in the apparatus). I found the presentation of results made it very difficult to separate the meaningful and more abundant types of cells from these very obscure ones. Some cells are likely to correlate with certain events merely by chance, how did the authors decide which populations should be included for analysis or represent statistically meaningful groups of cells? I strongly suggest applying a threshold or conducting some probability based analysis and then eliminate or move the presentation of the infrequent cell types (e.g. occurring in fewer than 3-4% of neurons) to a supplemental file or a figure only. The number of different and idiosyncratic labels for the types of cells makes the results section almost impossible to decipher. A minor point related to the presentation, the authors used just one letter to denote many aspects of their design, such as E=empty, O=object. While most of this is acceptable, with so many variations in the test, looking at the figures would be far more intuitive if whole words were used as labels rather than letters only. The consequence of these two points is evident in Figure 3 panels D, F H…it is impossible to make sense of these charts without adding copious additional notes to the panel, and all of this is probably wasted energy because many of these cell categories are simply not enough neurons to discuss in this brief report.*

We thank the reviewer for this constructive suggestion. In the previous version of our manuscript, we described all cell categories we identified, as we thought it was important and informative for readers. In addition, we adopted short or one-letter labels to represent some of the cell categories and experimental conditions to save figure space and character count of the text.

In response to this comment, as noted in our response to Reviewer #1’s comment above, we corrected the statistical significance levels for multiple comparisons and completely re-analyzed our data. This correction eliminated some of small cell categories that were likely identified by chance in the original analysis (e.g. “Wall cells” that constituted 1.2% of total cells in the original analysis) and allowed us to exclude these cells from our results. We also improved our data presentation by including less relevant cell categories to “Other cells” (Figs 2E and 4B; page 41, line 660–661; page 43, lines 697–699) and by moving some figures to Supplementary Figures (S1 Fig and S3 Fig). However, we have shown all cell categories in Fig 3D–F as we believe it is useful for comparing the cell fractions side-by-side between different sessions even if some categories contain no cells in some conditions.

Regarding the labels, we have eliminated one-letter labels and idiosyncratic labels as much as possible throughout the manuscript. For example, we have replaced the one-letter labels for the content of the chamber (i.e. “E” for empty, “S” for stranger and “O” for object) with the whole words indicated in the subscript in Fig 3 and S3 Fig and throughout the text (page 11, line 145).

*The discussion suggests the Kim et al 2015 paper was the basis for investigating the insula, however, there are many other relevant manuscripts indicating a role for insula in social behavior namely the Rogers-Carter et al 2018 paper that is mentioned in the introduction and also Gerlach et al 2019.

The discussion should begin with a concise account of the key findings. Instead it starts with discussing a relatively small group of social-off cells and then moves onto very speculative points about where these neurons might project in hypothetical circuits. For example, this sentence “The Social-ON and Social-OFF ensembles can potentially increase signal-to-noise ratio of information coding at a population level and can separately route opposing information via segregated projections to distinct downstream neurons.” Is completely speculative and not supported by the provided data or set in a discussion context that is meaningful (concepts like S/N & projection circuits are not introduced).*

*Line 228 of the discussion is possible incorrect. Insula has relatively few projects to PL in rat (the papers cited are rat, not mouse papers) with a heavy projection to IL. A quick pass at the Allen connectivity atlas for mouse dose show PL projections, but they are more sparse than ventral PFC areas and depend highly on the location of the tracer. The point here is that there is a lot of speculation about the circuits between AI and other regions, but the reviewed literature may be misrepresented, and the reported data offer no support for these track specific issues.

To address this, the authors should spend more time considering what type of information would be encoded by the different subsets of cells and what other findings about insular cortex these can relate to. The second paragraph of the discussion begins to do this, but again veers into speculation. Something that the authors might consider framing their cells against findings of others, for example, Gerhlach et al show clear role of this region in fear and anxiety expression, Rogers-Carter et al 2018, and recently 2019, show effects only on social emotional behavior but not sociality per se. In the third paragraph there discussion returns to a theme set up in the introduction regarding the salience network. It would be nice to see more analyses and discussion of how the neurons found in the insula might correlate with “salience” within this context.

Overall, these data are important, and it is worth the effort to revise this manuscript so that the key conclusions are more accessible to the reader and the discussion is focused on the inferences that can be drawn directly from the data rather than speculation. I also suggest the authors consider including the following recent publications that relate to this topic:

1: Rogers-Carter MM, Djerdjaj A, Gribbons KB, Varela JA, Christianson JP. Insular
Cortex Projections to Nucleus Accumbens Core Mediate Social Approach to Stressed
Juvenile Rats. J Neurosci. 2019 Oct 30;39(44):8717-8729. doi:
10.1523/JNEUROSCI.0316-19.2019. Epub 2019 Oct 7. PubMed PMID: 31591155; PubMed
Central PMCID: PMC6820210.

2: Rogers-Carter MM, Christianson JP. An insular view of the social
decision-making network. Neurosci Biobehav Rev. 2019 Aug;103:119-132. doi:
10.1016/j.neubiorev.2019.06.005. Epub 2019 Jun 10. Review. PubMed PMID: 31194999;
PubMed Central PMCID: PMC6699879.

3: Gehrlach DA, Dolensek N, Klein AS, Roy Chowdhury R, Matthys A, Junghänel M,
Gaitanos TN, Podgornik A, Black TD, Reddy Vaka N, Conzelmann KK, Gogolla N.
Aversive state processing in the posterior insular cortex. Nat Neurosci. 2019
Sep;22(9):1424-1437. doi: 10.1038/s41593-019-0469-1. Epub 2019 Aug 27. PubMed
PMID: 31455886.

4: Berret E, Kintscher M, Palchaudhuri S, Tang W, Osypenko D, Kochubey O,
Schneggenburger R. Insular cortex processes aversive somatosensory information
and is crucial for threat learning. Science. 2019 May 31;364(6443). pii:
eaaw0474. doi: 10.1126/science.aaw0474. Epub 2019 May 16. PubMed PMID: 31097492.*

Following this comment, we have thoroughly revised our Discussion. First, we removed our previous discussion regarding information on signal-to-noise ratio and hypothetical projection circuits from the text. We then summarized our key findings in the first paragraph as suggested (page 14, line 198–page 15, line 211), followed by the second paragraph in which we discuss what information is encoded in the AI in light of published literature, with particular emphasis on the function of anterior versus posterior ICs (page 15, line 212–page 16, line 230). In the third paragraph, we discuss the properties and possible function of Social cells in AI in relation to the function of “salience network” in humans (page 16, line 231–page 17, line 259).

We have cited the suggested publications #1–3 in Introduction and Discussion of the revised manuscript (Ref [18], [20] and [33]; page 5, lines 33 and 41–42; page 14, line 203; page 15, line 214, 216 and 217; page 33, lines 521–521 and 527–529; page 35, lines 559–560), although we did not cite the publication #4 because this paper was retracted before our revision.

*Reviewer #3: The authors have discovered Social-ON and Social-OFF cells intermingled in the agranular insular cortex (AI) and they showed that the Social-ON cells represented social experience independent of location and consisted of multiple subsets, each of which were preferentially active during exploration under particular behavioral state or with a particular target of physical contact.

Using a direct interaction they identified Social-ON cells (about 20% of total) and Social-OFF cells (about 3%), with some Social-ON cells exhibiting activity in specific parts of social interaction – such as stationary or moving, or contacting the nose, body or anogenital area.

Then they used a modified 3-chamber test to show again B(S)-ON cells (16%) more active in social nose poke and B(S)-OFF (4%) less active in social nose poke, with some overlap between the Social-ON vs B(S)-ON cells and Social-OFF vs B(S)-OFF cells.

In general the finding of the AI cells with activity is interesting, though the study seems somewhat preliminary and the second set of experiments with the 3-chamber test does not seem to add much to the initial finding. For example, it would have been more interesting to test whether female stimulus evokes similar response and whether those cells are overlapping. Also the choice to test AI is not particularly well justified and it would be interesting if similar cells are in other related cortices or if this is a unique feature to AI. Finally, multi-color retrograde tracers could have been used to test whether these cells have some common projections, which would strongly support their unique function.*

We thank the reviewer for this constructive comment. Our linear chamber (LC) experiments demonstrate that the increase in the fraction of Social cells occurs independent of the location and that neurons encoding spatial information *per se* are essentially absent in AI. We believe that these findings provide valuable insight into our understanding of the response properties of insular Social cells.

To address this comment, we analyzed a separate dataset we had already obtained from LC experiments using a female stranger mouse. The result indicated that, similar to a male stranger mouse, the presentation of a female stranger mouse increased the number of cells whose activity correlated with the investigation of the chamber in which the mouse was placed. We have presented this new finding in S4 Fig (S4 Fig; page 12, lines 163–164; page 22, lines 334–335; page 46, line 755–page 47, line 762). Our preliminary analysis also suggested that social cells identified in male LC experiments and female LC experiments overlapped very weakly (one overlapping cell out of 6 male-responsive cells [11.8% of total 51 cells] and 21 female-responsive cells [20.0% of total 105 cells] from 2 mice). However, drawing a firm conclusion requires a larger sample size and further additional control experiments. We would like to leave the rest of the suggested experiments for future investigations following this Short Report study. Instead, we have added a new sentence to the Discussion, which reads “*Thus, future studies such as identification and manipulation of the projection targets of Social-On and Social-OFF cells and comparison of their activity between anterior and posterior ICs in a socioemotional task will greatly advance our understanding of their significance and circuit mechanisms.*” (page 15, line 225–page 16, line 228).
