# Supplementary material for: Encoding of social exploration by neural ensembles in the insular cortex
Source: PLoS Biol. 2020 Sep 21;18(9):e3000584. doi: 10.1371/journal.pbio.3000584 (PMC7529241; doi:10.1371/journal.pbio.3000584)
Supplement: Attachment — Submitted filename: response_200718.docx [file pbio.3000584.s010.docx]

**Point-by-point responses to the reviewers’ comments**

*Reviewer #2: In this revised manuscript the authors have improved from the original but the report still has room for improvement. Briefly, the authors used miniscopes and calcium imaging in the anterior insular cortex of mice performing either a social interaction task, or a linear box sociability task with a novel object or a novel conspecific at either end. In the social interaction test, ~22% of recorded cells increased firing to social interaction and of these cells some exhibited preferential firing for specific conspecific body parts. This is probably the most exciting and new piece of information in the report. In the linear sociability task, the largest portion of cells identified increased firing to the novel mouse, but only a few (~3%) of these cells were consistently "social." The authors make a number of inferences from these observations about the nature of social encoding in anterior insula. While the inferences may eventually be determined accurate, at this
point, without mechanistic tests (i.e. loss of function study of these specific cells) the authors must broaden the range of inferences and specifically suggest alternatives to a strictly social account. It seems that the manuscript, and perhaps, some of the author's inferences could be tested with additional graph analysis of the putative neuronal ensembles discovered.*

*Ln 88. It is distracting to mention the small fraction of movement and stationary cells and then return to discuss the Social-ON cells that correlated with specific interaction types. In this reviewer's opinion, they should be moved to a supplementary section or not mentioned at all as they represent such a small fraction of the recorded cells (about 5%).*

Following this suggestion, we have removed the sentences and figures (former S2A–B Fig) that referred to Movement cells and Stationary cells. We have mentioned these cell categories as a part of “other cells” only in Fig. 2E legend (page 10, lines 130–133).

*Ln 107. The support for this statement is not robust. The authors appear to be using that some cells that increase firing during social interaction also encode behavioral state and target of contact. I suggest tempering this inference or conducting more analyses to establish "conjunctive information." All of the categories are defined by human observers, what the firing actually encodes to the mouse could be quite different (and may not be "conjunctive").*

Following this comment, we have tempered our statement by removing the word “conjunctive” from the sentence (page 12, line 159). We have removed the same word from the Abstract as well (page 3, lines 11–14).

*Ln 178. Only 3.6% of cells initially defined as Social cells were consistent across sessions. The authors seem to interpret this as a significant amount. However, if the insula is actually a salience detector and sight of sensory integration, one might actually predict that cells don't have unitary types of encoding that would be seen in sensory systems or hippocampal place cells. Simply tallying these groups of cells seems is an underpowered approach to analysis and so the inferences made may also be too simplistic. Did the authors consider functional connectivity/graph network analysis of activity among cells. A fascinating possibility is that over sessions, the same sort of information (social or not) is encoded within the insula, but the specific cells that carry such information changes. This occurs in the circuits that orchestrate birdsong—while bird songs are consistent, the cells that "sing" the song are networked together such that a given note (or bit of information) may be played by any one of several interconnected neurons that, once fired, will trigger the next network node and the song continues. Given the vast number of circumstances in which insular cortex increases activity (across rodent and human studies) it seems more likely that these social cells are actually a sign of some other type of encoding (salience is one possibility) that is not specific to task, but might change depending on internal states, past experience, etc. For the amount of data available from the miniscope investigation, the analysis and working model that the investigators present remains under developed.*

We thank the reviewer for this insightful comment. First of all, during the course of addressing this comment, we found that five out of 19 consistent Social-ON cells responded not only to the investigation of the chamber containing a mouse (i.e. chamber A) but also to the other chamber in the second interaction sessions. Although these cells could be interpreted to encode the past and present history of social investigation, we have rigorously excluded these cells from consistent Social-ON cells and have updated the relevant data in the text and figures (page 17, lines 250–255; Figure 4B–D). Importantly, we confirmed that these changes did not affect our conclusion that the observed number of consistent Social cells is significantly higher than that is expected by chance (Figure 4D). This finding implies that the existence of consistent Social cells is a significant phenomenon.

Second of all, our data in Figure 4B means that 2.8% (previously 3.6%) of total cells, but not of initial Social cells as the reviewer noted, consistently responded to social investigation. This fraction is equivalent to 19.7% of Social-ON cells (14 out of 71 cells) and 22.2% of Social-OFF cells (two out of nine cells) in the first interaction sessions, which we think are not negligibly small. To avoid this confusion, we have included these fractions in the text (page 17, lines 250–251).

Finally, to gain insight into the functional connectivity among Social-ON neurons, we calculated pair-wise correlation of Social-On cell activity during social investigation periods in the first interaction sessions. The average correlation coefficient of activity of consistent Social-ON cells did not significantly differ from that of the cells that were Social-ON cells only in the first interaction sessions (consistent Social-On cells, 0.103 ± 0.090 vs Social-ON cells in the first interaction session only, 0.127 ± 0.083; mean ± SD, p = 0.32, t_(377)_ = 1.00, unpaired t test, n = 12 and 367 cell pairs from 6 mice). These results suggest that it is unlikely that consistent Social-ON cells are more strongly functionally connected with each other than the rest of Social-ON cells. In addition, their low levels of activity correlation imply that Social-ON cells generally rarely show synchronized activity during social investigation and make further functional connectivity analysis difficult to be conducted and interpreted.

*Ln 210. This inference is one of many (that insula neurons encode the status of social exploration). An alternative account already has more support in the literature which is that insula encodes the internal states/values (see Linveh et al recent papers). It is likely that as social interaction progresses, the focal test mouse state changes. There is a challenge to interpreting social cells because it is very difficult to untangle whether the cells encode the "other" or if they encode something evoked by the other within the "self."*

In light of this comment, we have added new sentences to the relevant part of the Discussion, which now reads “These findings imply that neuronal ensembles in AI encode the ongoing status of social exploration while an animal interacts with unfamiliar conspecifics. However, insular neurons are also shown to be able to encode internal states independent of behavior [38]. It thus remains to be investigated whether Social cells in AI encode interaction with "other" and/or changes in the state of “self” associated with social exploration.” (page 19 lines 298–301).

ETHICS STATEMENT:

*Please indicate within the manuscript, explicitly, the name of the national or international ethical guidelines to which your experimental protocols approved by the RIKEN Animal Experiments Committee adhered.*

We have included the relevant information in the text (page 23, lines 353–356).

*Please provide within the manuscript the ID number of the protocols approved by the RIKEN Animal Experiments Committee.*

We have included the relevant information in the text (page 23, line 354).

FINANCIAL DISCLOSURE:

*Please update your financial disclosure in the submission system to indicate the sources of funding that *directly* supported this work.*

We have updated our financial disclosure in the submission system accordingly.

DATA POLICY:

*Please update your S1 Data File to include data for figure 2F and S1B.*

We have included data for Figure 2F and S1B in our S1 Data File.

*Please ensure that the figure legends in your manuscript include information on where the underlying data can be found.*

We have included the relevant information in the figure legends.
